# Global research trends and hot spots on autophagy and kidney diseases: a bibliometric analysis from 2000 to 2022

**DOI:** 10.3389/fphar.2023.1275792

**Published:** 2023-11-30

**Authors:** Sinan Ai, Yake Li, Huijuan Zheng, Zhen Wang, Weijing Liu, JiaYin Tao, Yaotan Li, Yaoxian Wang

**Affiliations:** ^1^ Beijing University of Chinese Medicine, Beijing, China; ^2^ Dongzhimen Hospital, Beijing University of Chinese Medicine, Beijing, China; ^3^ Beijing Hospital of Traditional Chinese Medicine, Capital Medical University, Beijing, China; ^4^ Key Laboratory of Chinese Internal Medicine of Ministry of Education and Beijing, Dongzhimen Hospital Affiliated to Beijing University of Chinese Medicine, Beijing, China; ^5^ Henan University of Chinese Medicine, Zhengzhou, China

**Keywords:** autophagy, kidney disease, kidney, bibliometric analysis, hotspots, trends

## Abstract

**Background:** Autophagy is an essential cellular process involving the self-degradation and recycling of organelles, proteins, and cellular debris. Recent research has shown that autophagy plays a significant role in the occurrence and development of kidney diseases. However, there is a lack of bibliometric analysis regarding the relationship between autophagy and kidney diseases.

**Methods:** A bibliometric analysis was conducted by searching for literature related to autophagy and kidney diseases in the Web of Science Core Collection (WoSCC) database from 2000 to 2022. Data processing was carried out using R package “Bibliometrix”, VOSviewers, and CiteSpace.

**Results:** A total of 4,579 articles related to autophagy and kidney diseases were collected from various countries. China and the United States were the main countries contributing to the publications. The number of publications in this field showed a year-on-year increasing trend, with open-access journals playing a major role in driving the literature output. Nanjing Medical University in China, Osaka University in Japan, and the University of Pittsburgh in the United States were the main research institutions. The journal “International journal of molecular sciences” had the highest number of publications, while “Autophagy” was the most influential journal in the field. These articles were authored by 18,583 individuals, with Dong, Zheng; Koya, Daisuke; and Kume, Shinji being the most prolific authors, and Dong, Zheng being the most frequently co-cited author. Research on autophagy mainly focused on diabetic kidney diseases, acute kidney injury, and chronic kidney disease. “Autophagy”, “apoptosis”, and “oxidative stress” were the primary research hotspots. Topics such as “diabetic kidney diseases”, “sepsis”, “ferroptosis”, “nrf2”, “hypertension” and “pi3k” may represent potential future development trends. Research on autophagy has gradually focused on metabolic-related kidney diseases such as diabetic nephropathy and hypertension. Additionally, PI3K, NRF2, and ferroptosis have been recent research directions in the field of autophagy mechanisms.

**Conclusion:** This is the first comprehensive bibliometric study summarizing the relationship between autophagy and kidney diseases. The findings aid in identifying recent research frontiers and hot topics, providing valuable references for scholars investigating the role of autophagy in kidney diseases.

## 1 Introduction

Autophagy is a fundamental cellular process that degrades and recycles damaged or expired cellular organelles, proteins, and other cellular components, maintaining cellular function and organism homeostasis (Mizushima and Komatsu, 2011; Xiao et al., 2017; Tang et al., 2018). Over the past few decades, research has uncovered a close association between autophagy and the development and progression of various disorders, including kidney diseases (Mizushima et al., 2008; Rubinsztein et al., 2012). The procedure of autophagy involves multiple stages, including the formation and maturation of autophagosomes, their fusion with lysosomes for degradation, and releasing into the cytoplasm for recycle (Yin et al., 2016). In 2016, Yoshinori Ohsumi was awarded the Nobel Prize in Physiology or Medicine for breakthrough discoveries in the biological process of autophagy, driving advancements in biomedical research (Levine and Klionsky, 2017). The core signaling pathways of autophagy, such as mammalian target of rapamycin (mTOR), AMP-activated protein kinase (AMPK), and Sirtuins (SIRTs), play crucial roles in regulating the autophagic process. Dysregulation of these signaling pathways may result in impaired autophagy, which is closely associated to the occurrence and progression of kidney diseases (Yin et al., 2016). However, autophagy has two-sided impact in kidney diseases. On one hand, autophagy acts as a protective mechanism by clearing aberrant proteins and dysfunctional organelles, thereby maintaining normal renal cell function. Thus impaired autophagy can trigger pathological processes such as inflammation, cell apoptosis, and fibrosis, contributing to the progression of diverse kidney diseases. On the other hand, under certain conditions, excessive or dysregulated autophagy may contribute to disease pathogenesis and cell death.

Autophagy plays distinct roles in various kidney diseases (Choi, 2020). In acute kidney injury (AKI), autophagy act as regulator of the injury and death of tubular epithelial cells (Periyasamy-Thandavan et al., 2008). In chronic kidney disease (CKD), decreased autophagy function may lead to renal fibrosis (Ding et al., 2014). In diabetic kidney disease (DKD), abnormal autophagy is associated with an imbalance in energy metabolism of renal cells in a hyperglycemic environment (Kitada et al., 2017). Additionally, autophagy is also related to polycystic kidney disease and kidney cancer (Liu et al., 2015; Zhu et al., 2017; Lee et al., 2020). Modulation of autophagy signaling pathways has arisen as a potential therapeutic approach for kidney diseases. For instance, metformin promotes autophagy occurrence by stimulating the AMPK signaling pathway or targeting mTOR complex 1 (mTORC1) inhibitors, thereby alleviating the pathological progression of kidney diseases (Song et al., 2021). While rapamycin, an autophagy inducer, may acts as therapeutic capacity to protect kidneys from injury (Su et al., 2018; Namwanje et al., 2021). Furthermore, certain natural compounds and herb medicine have been demonstrated to modulate autophagy, offering potential for the treatment of kidney diseases (Wang et al., 2019). Gaining a comprehensive understanding of the interplay between autophagy and kidney diseases is crucial for unraveling their underlying mechanisms and establishing potential therapeutic approaches. Thus, further exploration and validation are essential to elucidate the precise roles and regulatory mechanisms of autophagy in kidney diseases.

In the past 2 decades, a substantial body of evidence has confirmed the vital role of autophagy in kidney disease progression, and numerous recent reviews have deeply explored various facets of this theory (Choi, 2020; Tang et al., 2020). However, there is currently a lack of thorough analysis regarding the present state and upcoming directions, as well as objective descriptions that highlight the research focus in the area. Bibliometrics, introduced by Alan Pritchard in 1969, is a statistical approach that analyzes existing literature to identify development trends and research hotspots in a specific field (Hassan et al., 2021; Ninkov et al., 2022). This method has been widely utilized in diverse disciplines, including medical research (Cárdenas-Figueroa and Navarro, 2020; Nobanee, 2021; Wilson et al., 2021). By conducting bibliometric analyses of publications, countries, institutions, journals, authors, and their co-occurrences, a comprehensive understanding of the developmental process in the research field can be obtained. Additionally, to identify the basis, frontiers, and highlights of the field, we will utilize author keywords and conduct keyword co-occurrence analyses. To our knowledge, bibliometric research has been conducted on the relationship between autophagy and cardiovascular diseases, osteoarthritis, cancer, and lung diseases (Zhou et al., 2021; Lin et al., 2022; Xu et al., 2022; Liao et al., 2023), but it has not been applied to the study of autophagy and kidney diseases. Therefore, our purpose is to uncover the existing research status and future directions of autophagy in kidney diseases through bibliometric analysis, identifying major contributors, institutions, countries, and the current research focus, to provide a reference for future studies on autophagy in kidney diseases.

## 2 Materials and methods

### 2.1 Data source and search strategy

**FIGURE 1:**
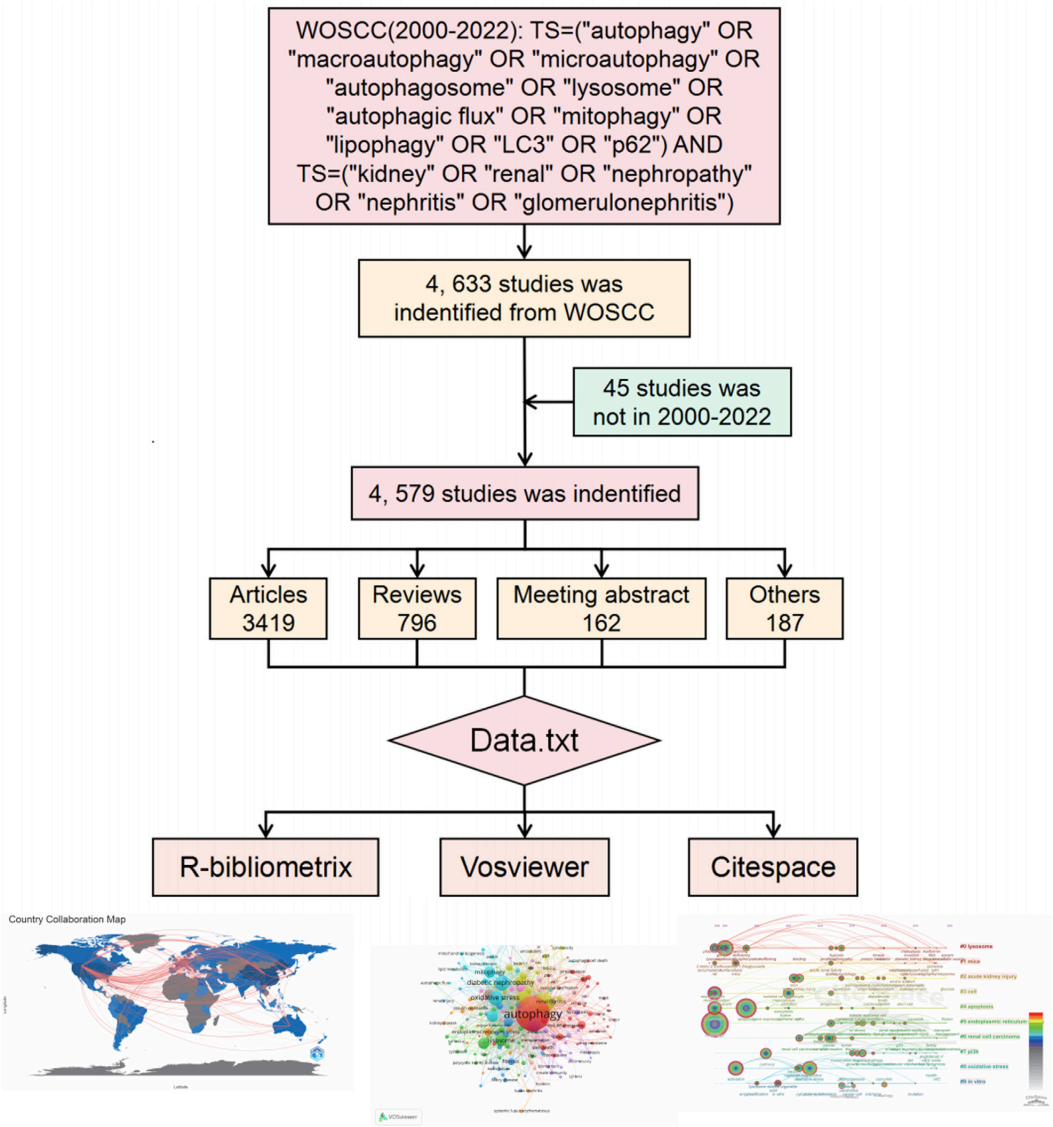
The flowchart of bibliometric analysis in autophagy and kidney disease.

We performed an extensive search on the Web of Science Core Collection (WoSCC) database for performed analysis, covering English literature from the year 2000–2022 (up until 30 December 2022). Bibliometric analysis is commonly carried out using both WoSCC and Scopus databases (Zhu and Liu, 2020), and we selected WoSCC because of its extensive coverage of reputable and influential journals, which is highly favored by researchers in the academic community. We utilized the search term combination TS= (“autophagy” OR “macroautophagy” OR “microautophagy” OR “autophagosome” OR “lysosome” OR “autophagic flux” OR “mitophagy” OR “lipophagy” OR “LC3” OR “p62”) AND TS= (“kidney” OR “renal” OR “nephropathy” OR “nephritis” OR “glomerulonephritis”) to collect all relevant records. The search period spanned from 2000 to 2022. Although there were already a considerable number of articles on autophagy before 2000, there were relatively fewer articles specifically emphasizing the correlation between autophagy and kidney diseases. Therefore, we considered this timeframe to be representative of the evolving patterns in our area of interest. All relevant documents concerning autophagy and kidney diseases were exported as “full records and references” in TXT format. The literature was first imported into Citespace for de-duplication processing. Subsequently, in Excel, the literature that was not within this time period was removed. They were then retrieved and brought in the R package “bibliometrix”, VOSviewer, and CiteSpace for comprehensive bibliometric analysis. The workflow diagram is presented in Figure 1.

### 2.2 Data analysis

Bibliometric tools including R package “bibliometrix”, VOSviewer, CiteSpace are commonly used for visualizing the results of bibliographic analysis (Wu et al., 2022; Liao et al., 2023). And we selected appropriate software to analyze different aspects of our research results for optimal presentation. In this research, we employed R (version 4.1.2), R-bibliometrix package (version 3.0.3, http://www.bibliometrix.org), CiteSpace (version 6.1. R2), and VOSviewer software (version 1.6.18) for visual analysis of the selected literature (Agnusdei and Coluccia, 2022). Using these tools, we analyzed the publications, countries, institutions, journals, authors, and keywords. We utilized Biblioshiny, a user-friendly web interface for R-bibliometrix, to conduct visual display of data analysis and social network graphs. And we enhanced the visualization of our findings applying the powerful R package ggplot2 (version 3.4.2). We utilized the R-bibliometrix package to perform frequency statistics on authors, journals, institutions, and countries, including publication counts and citation numbers. We displayed the top 20 frequencies, and in the word cloud for the top 50 author keywords, the size of the words represents their frequency of occurrence. The remaining software parameters were set to their default values. Furthermore, we employed VOSviewer software (version 1.6.16, https://www.VOSviewer.com/download), developed by Nees Jan van Eck and Ludo Waltman from Leiden University, to generate core authors, countries, research institutions, and keyword co-occurrence networks, where the size of nodes represents the frequency of appearance or citation and the thickness of the connecting lines between nodes indicates the strength of collaboration. In the keyword co-occurrence network, we set the minimum frequency for authors, institutions, journals, and keyword appearances to be 10. For countries, the minimum appearance frequency was set at 5, and for article citation counts, the minimum frequency was set to 100. In addition, another commonly used software for bibliometric analysis and visualization is CiteSpace, which is created by Professor Chaomei Chen. In our research, CiteSpace was applied to perform words burst analysis and to visualize the dual-map overlay. We conducted keyword K-means cluster analysis with a G-index value of k = 25, using Pathfinder for pruning sliced networks and merged networks. Additionally, we applied the software’s Timeline View and Burstness features for keyword timeline graphs and keyword burst analysis, with default parameter settings.

## 3 Results

### 3.1 Number of publications and citation evolution

**FIGURE 2:**
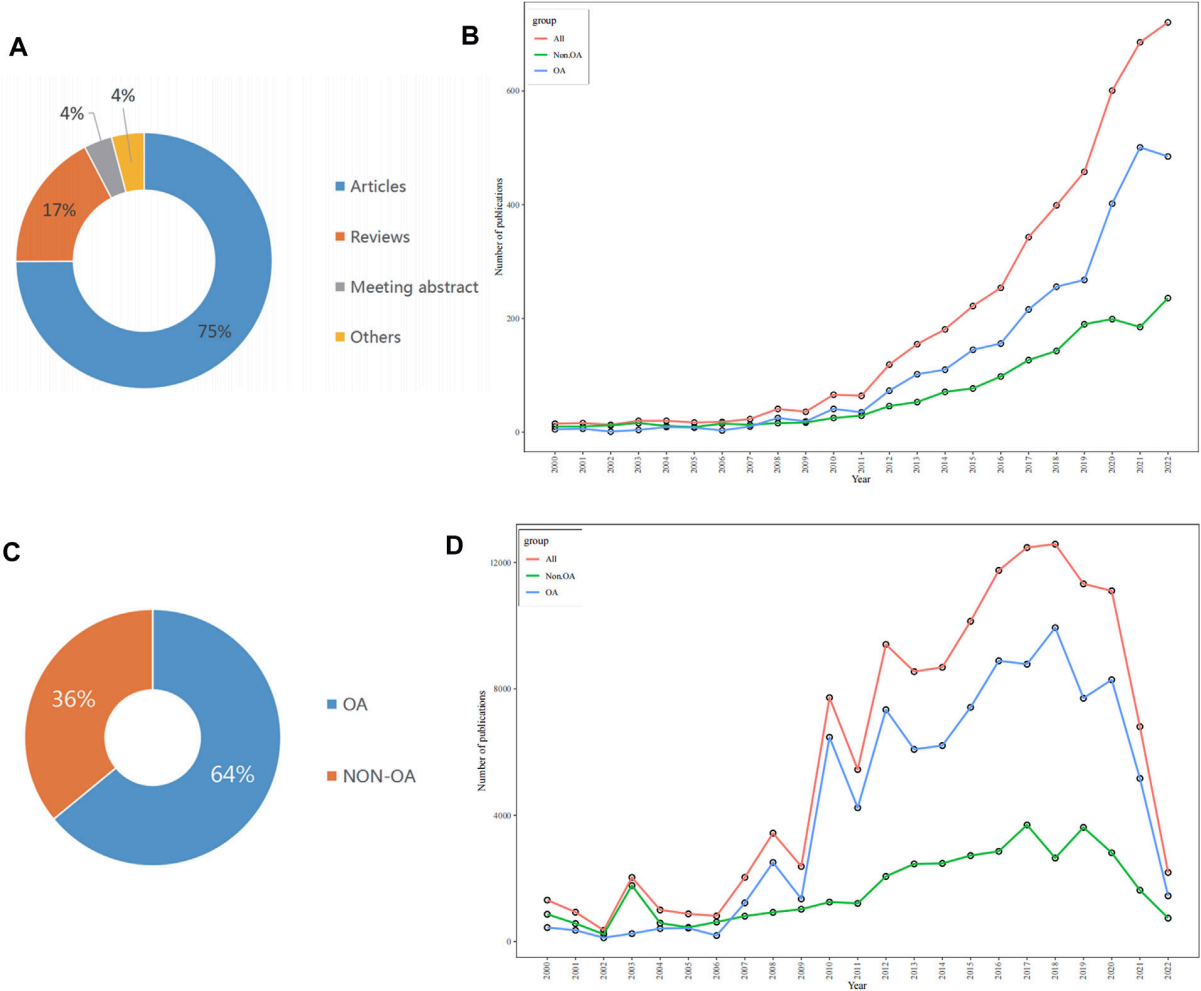
Analysis of publications in autophay and kidney disease by using bibliometric. **(A)** Proportion of publication types. **(B)** Trends in annual publications. **(C)** Proportion of open non-open access (non-OA) and open access (OA) publications. **(D)** Trends in annual total citations.

A total of 4,579 publications were taken into account in this study from the Web of Science Core Collection (WoSCC) database, comprising 3,419 articles, 796 reviews, 162 conference abstracts, and 187 other literature types, including book chapters, letters, or editorials (Figure 2A). A line graph illustrating the changing quantity of literature on autophagy and kidney diseases from 2000 to 2022 was created (Figure 2B). The entire period can be categorized into three phases based on the yearly growth rate of publications: the first phase (2000–2011), the second phase (2012–2016), and the third phase (2016–2022). From 2000 to 2011, the number of published articles remained relatively low. Although there were already a significant number of articles related to autophagy during this period, only a few researchers focused on studying the correlation between autophagy and kidney diseases. Since 2012, there has been a gradual increase in the number of published articles, with an increasing trend each year. After the autophagy research was awarded the Nobel Prize in 2016, the number of publication has experienced an explosive growth. A total of 3,462 articles were released from 2016 to 2022, accounting for 75.61% of all documents. The data suggests a rising scholarly interest in the study of autophagy and its relation to kidney diseases in recent years. Besides, a subgroup analysis was performed of publications based on non-open access (non-OA) and open access (OA) documents. Among the 4,579 articles, 1,618 (35.34%) were detected as non-OA, whereas 2,961 (64.66%) were OA (Figure 2C). Based on our observations, the growth rate of OA articles (23.38%) surpassed that of non-OA articles (15.71%), in Figure 2B. Furthermore, the overall citation count for all 4,579 articles was 137,690, with an average of 99,140 citations for OA articles and 38,508 citations for non-OA articles. The citation counts consistently showed lower numbers for non-OA articles, and the overall citation trend closely followed the pattern observed for open access articles (Figure 2D), which indicates that open-access journals have a significant impact on driving advancements in this research area.

According to the co-citation network analysis VOSviewer (Supplementary Figure S1A), the article with the highest number of citations in the WoSCC database is authored by Eileen White. The article titled “Deconvoluting the context-dependent role for autophagy in cancer” was released in Nature Reviews Cancer (IF = 69.8) in 2012 and has accumulated a total of 1,254 citations (White, 2012). The second most highly cited article is “Termination of autophagy and reformation of lysosomes regulated by mTOR” by Li Yu, published in Nature (IF = 69.504) (Yu et al., 2010). Table 1 lists the top 20 most highly cited articles according to Biblioshiny. Subsequently, we constructed a timeline arranged in chronological order to highlight the important milestones characterized by these significant articles by assessing the citation count and the relevance to autophagy and kidney diseases (Supplementary Figure S1B).

**TABLE 1:** Top 20 most cited articles.

Title	First author	Journal	Year	Total citations	If	JCR Partition
Deconvoluting the context-dependent role for autophagy in cancer	Eileen White	Nat Rev Cancer	2012	1,261	78.5	Q1
Termination of autophagy and reformation of lysosomes regulated by mTOR	Li Yu	Nature	2010	1,079	64.8	Q1
From Krebs to clinic: glutamine metabolism to cancer therapy	Luigi Ferrucci	Nat rev cancer	2018	1,059	49.421	Q1
Early *versus* late parenteral nutrition in critically ill adults	Michael P Casaer	New engl j med	2011	984	158.5	Q1
Chloroquine inhibits autophagic flux by decreasing autophagosome-lysosome fusion	Mario Mauthe	Autophagy	2018	966	13.3	Q1
ERK and cell death: mechanisms of ERK-induced cell death--apoptosis, autophagy and senescence	Sebastien Cagnol	Febs j	2010	926	5.4	Q2
Accumulation of autophagic vacuoles and cardiomyopathy in LAMP-2-deficient mice	Y Tanaka	Nature	2000	695	64.8	Q1
Pesticides and human chronic diseases: evidences, mechanisms, and perspectives	Sara Mostafalou	Toxicol appl pharm	2013	677	3.8	Q2
Cellular death, reactive oxygen species (ROS) and diabetic complications	Caroline Maria Oliveira Volpe	Cell death dis	2018	582	9.0	Q1
Autophagy influences glomerular disease susceptibility and maintains podocyte homeostasis in aging mice	Björn Hartleben	J clin invest	2010	518	15.9	Q1
Fabry disease, an under-recognized multisystemic disorder: expert recommendations for diagnosis, management, and enzyme replacement therapy	Robert J Desnick	Ann intern med	2003	510	39.2	Q1
Pharmacological modulation of autophagy: therapeutic potential and persisting obstacles	Lorenzo Galluzzi	Nat rev drug discov	2017	507	120.1	Q1
A unified theory of sepsis-induced acute kidney injury: inflammation, microcirculatory dysfunction, bioenergetics, and the tubular cell adaptation to injury	Hernando Gomez	Shock	2014	491	3.1	Q1
SREBP-regulated lipid metabolism: convergent physiology - divergent pathophysiology	Hitoshi Shimano	Nat rev endocrinol	2017	484	14.978	Q1
At the acidic edge: emerging functions for lysosomal membrane proteins	Eeva-Liisa Eskelinen	Trends cell biol	2003	477	19.0	Q2
3D tomography reveals connections between the phagophore and endoplasmic reticulum	Päivi Ylä-Anttila	Autophagy	2009	474	13.3	Q1
Calorie restriction enhances cell adaptation to hypoxia through Sirt1-dependent mitochondrial autophagy in mouse aged kidney	Shinji Kume	J clin invest	2010	465	15.9	Q1
*Listeria* monocytogenes promotes tumor growth via tumor cell toll-like receptor 2 signaling	Bo Huang	Cancer res	2007	456	11.2	Q1
RAGE (Receptor for Advanced Glycation Endproducts), RAGE ligands, and their role in cancer and inflammation	Louis J Sparvero	J transl med	2009	442	7.4	Q1

### 3.2 Core journals analysis

**FIGURE 3:**
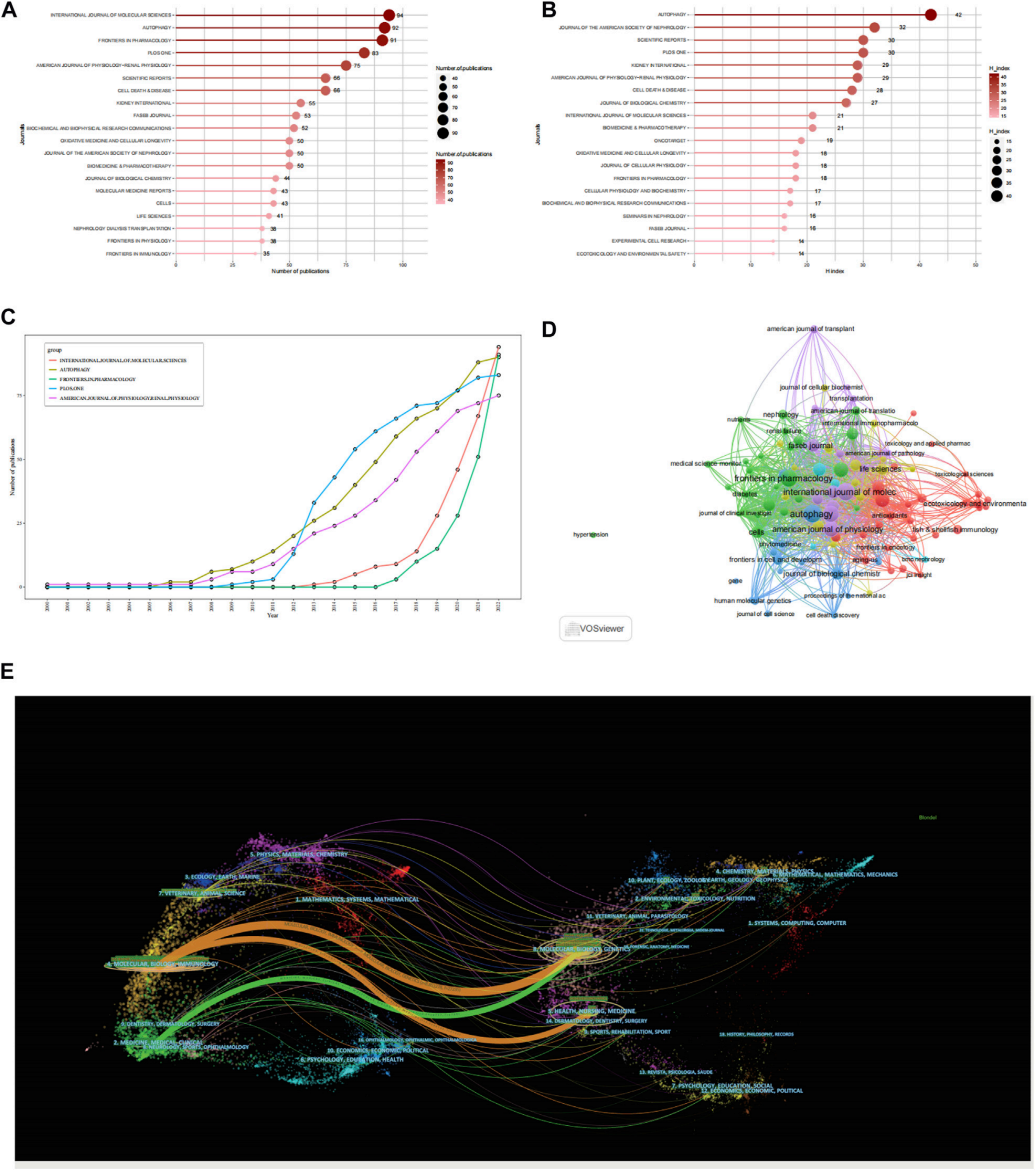
Analysis of journals. **(A)** Top 20 most productive journals contributing to research by using R-bibliometric. **(B)** Top 20 journals impact ranked by H-index by using R-bibliometric. **(C)** Top 5 journals publications trends overtime by using R-bibliometric. **(D)** Co-citation network of journals by using VOSviewer. **(E)** A dual-map overlay of journals by using citespace.

Figure 3A presents the 20 leading journals that published articles related to autophagy and kidney diseases between 2000 and 2022. Out of the 1,083 identified journals, 686 were non-OA journals and 587 were OA journals. The top 20 journals collectively published 1,157 documents, making up 25.27% of the total publications. Among them, the journal International journal of molecular sciences, holding an impact factor of 6.208 and Journal Citation Reports (JCR) Q1 ranking, released 94 relevant papers, contributing to 2.05% of all publications. Followed by FRONTIERS IN PHARMACOLOGY with 91 articles (1.99%), AUTOPHAGY with 90 articles (1.97%), PLOS ONE with 83 articles (1.81%), AMERICAN JOURNAL OF PHYSIOLOGY-RENAL PHYSIOLOGY with 75 articles (1.63%). The H-index is an indicator applied to assess the quantity and influence of journals (Hirsch, 2005; Engqvist and Frommen, 2008). Based on Figure 3B and Table 2, the two most influential journals, as indicated by the H-index, are Autophagy (H-index = 42) and Journal of the american society of nephrology (H-index = 32). Both are highly influential journals in the fields of autophagy and kidney diseases. Scientific reports and plos one are ranked third with an h-index of 30. kidney international and american journal of physiology-renal physiology, two nephrology journals, with an H-index of 29, rank fifth. The co-citation network of journals based on VOSviewer visualization (Figure 3D), also indicates that The three most prominent journals with the highest co-citations are autophagy, journal of the american society of nephrology, and kidney international.

**TABLE 2:** Top 20 most productive journals.

Rank	Journals	Documents	Impact factor	JCR Partition	H-index
1	International journal of molecular sciences	94	5.6	Q1	21
2	Frontiers in pharmacology	91	5.6	Q1	19
3	Autophagy	90	13.3	Q1	42
4	Plos one	83	3.7	Q2	30
5	American journal of physiology-renal physiology	75	4.2	Q1	30
6	Cell death & disease	66	9	Q1	29
7	Scientific reports	66	4.6	Q2	30
8	Kidney international	55	19.6	Q1	29
9	Faseb journal	53	4.8	Q2	16
10	Biochemical and biophysical research communications	52	3.1	Q3	17
11	Biomedicine & pharmacotherapy	50	7.5	Q1	22
12	Journal of the american society of nephrology	50	13.6	Q1	32
13	Oxidative medicine and cellular longevity	50	7.31	Q2	19
14	Journal of biological chemistry	44	4.8	Q2	27
15	Cells	43	6.0	Q2	13
16	Molecular medicine reports	43	Q3	3.4	13
17	Life sciences	41	6.1	Q1	15
18	Frontiers in physiology	38	4.0	Q2	15
19	Nephrology dialysis transplantation	38	6.1	Q1	10
20	Frontiers in immunology	35	7.3	Q1	11

According to the growth patterns of the leading 5 journals, as shown in Figure 3C, autophagy, frontiers in pharmacology, and american journal of physiology-renal physiology have consistently been popular journals within the area, maintaining a consistent increase in the number of published articles. They have shown a high sensitivity to autophagy research in kidney disease field, with relevant articles being reported as early as 2008. On the other hand, plos one and international journal of molecular sciences had fewer publications in the early stages but experienced rapid growth later on.

CiteSpace was utilized to assist in constructing the thematic distribution of academic journals (Figure 3E). Based on the overlay of journal co-occurrence maps, the journals publishing the articles primarily belong to the fields of molecular, biology, immunology, medicine, medical, and clinical. The articles with many citations were published in journals related to molecular, biology, genetics, health, nursing, and medicine.

### 3.3 Analysis of countries and institutions

**FIGURE 4:**
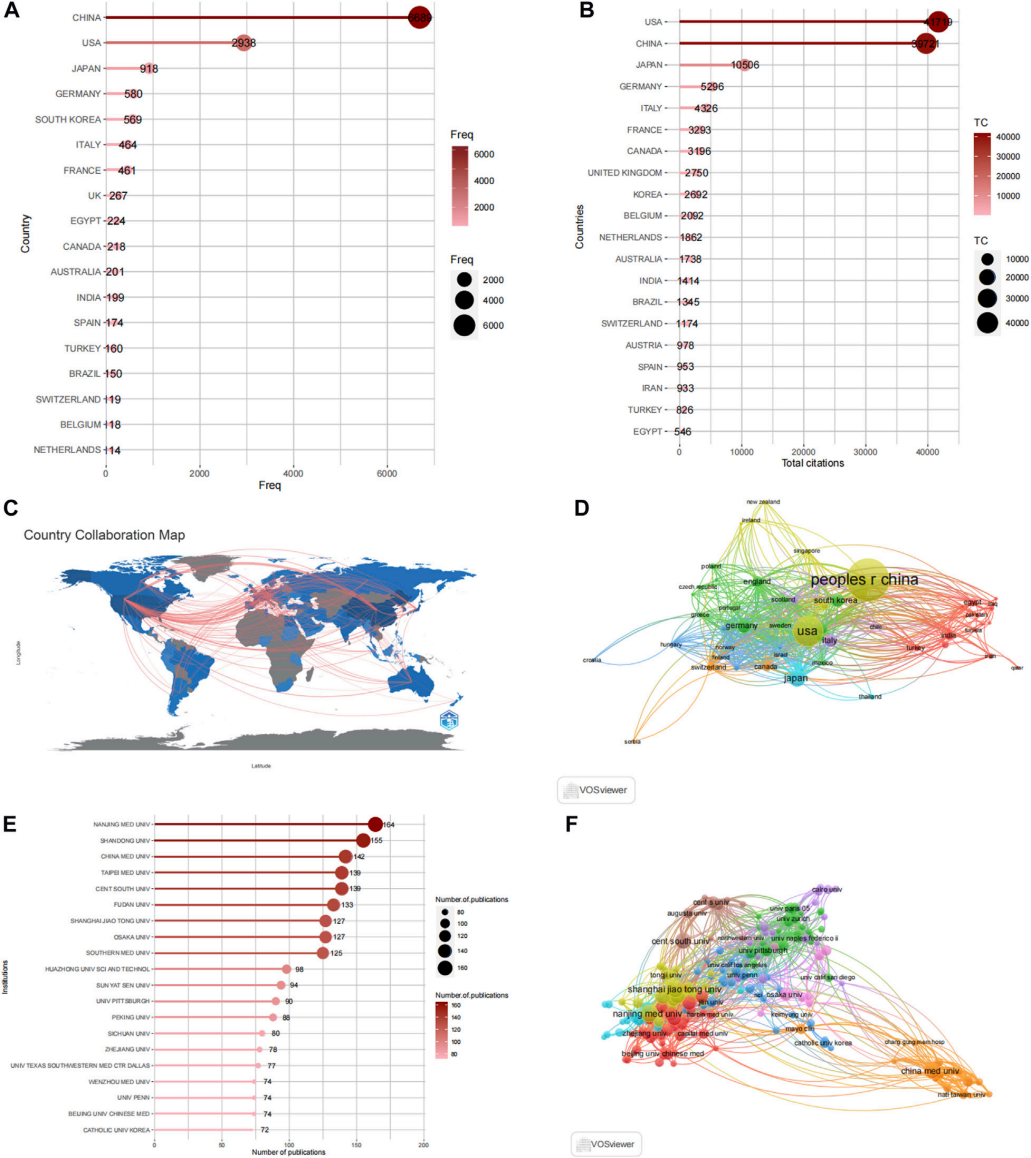
Analysis of countries and institutions. **(A)** Top 20 productive countries by using R-bibliometric. **(B)** Top 20 Most cited countries by using R-bibliometric. **(C)** Countries contribution and collaboration by using R-bibliometric. **(D)** Countries contribution and collaboration by using VOSviewer. **(E)** Top 20 productive institutions. **(F)** Institutions contribution and collaboration by using VOSviewer.

Figures 4A, B display the number of published articles and collaboration networks involving 74 countries researching autophagy and kidney diseases. China leads in productivity with 2,168 articles, followed by the United States (744 articles), Japan (252 articles), South Korea (167 articles), and Germany (120 articles). The United States and China are prominent Contributors in this research field, evident from their respective citation counts of 39,721 and 41,719, surpassing other countries like Japan (10,506), South Korea (5,296), and Italy (4,326). China’s rapid progress in the field of autophagy and kidney diseases is increasing its influence through closer collaborations and innovative research with other countries. Figures 4C, D depicts the collaboration relationships among the top-ranked countries by using VOSviewer and R-bibliometric, showing strong collaboration and exchanges primarily between the United States, China, and Western European countries. Among the 93 countries involved in international collaborations, the United States has the highest number of collaborations with other countries, totaling 530, followed by China with 424 collaborations.

**FIGURE 5:**
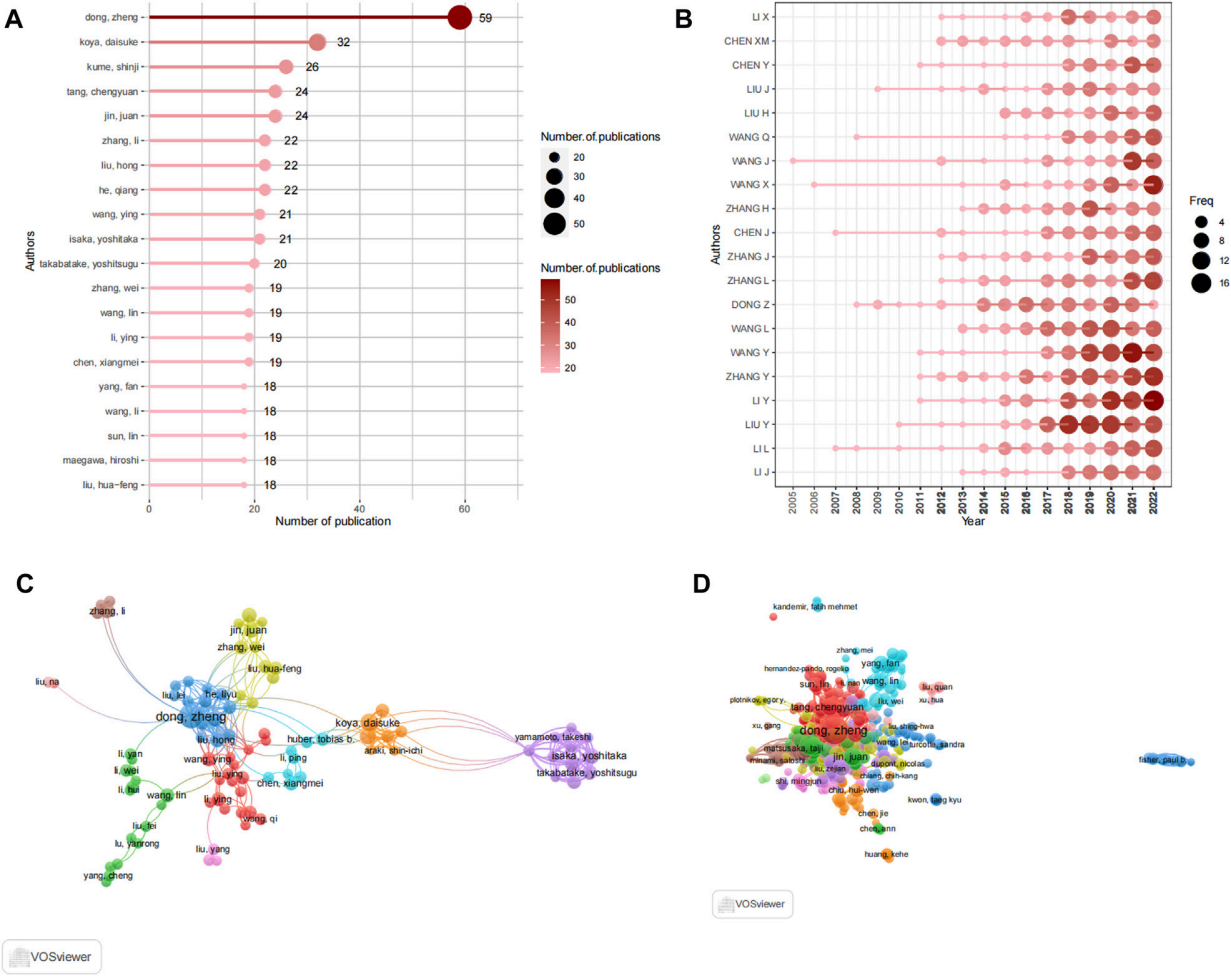
Analysis of authors. **(A)** Top 20 productive authors by using R-bibliometric. **(B)** Top 20 author's production over time by using R-bibliometric. **(C)** The visualization map author's collaboration network by using VOSviewer. **(D)** Co-citation network of authors by using VOSviewer.

These published articles originate from 3,768 institutions worldwide. The top 20 high-productivity institutions in autophagy and kidney disease research are presented in Figure 4E, with 75% (15 institutions) located in China. The remaining 25% (5 institutions) are situated in the United States (2 institutions), Japan (1 institution), Germany (1 institution), and South Korea (1 institution). Nanjing Medical University [has the highest number of published articles, with 164, followed by Shandong University (155 articles), China Medical University (142 articles), Taipei Medical University (139 articles), and Fudan University (139 articles). The collaboration network in Figure 5F shows the most influential institutions is China Medical University (109 total links), Charlie Norwood Veterans Affairs Medical Center (100 total links), and Fudan University (79 total links).

### 3.4 Contributions of authors

A total of 18,583 authors participated in contributing to the literature on autophagy and kidney diseases, resulting in an average of 4.12 authors per article. The top 20 most prolific authors collectively contributed to 1,005 publications (Figure 5A), representing 22.27% of all published works. Among these authors, Dong, Zheng from Xiangya Second Hospital, Central South University in China stood out with the highest number of publications, contributing 56 articles to the field. In 2020, an article titled “Autophagy in kidney homeostasis and disease” published in Nature Reviews Nephrology showcased the diverse roles of autophagy in kidney diseases (Tang et al., 2020), and it was cited 103 times. Following closely were Koya, Daisuke (32 articles), Kume, Shinji (26 articles), and jin, juan (24 articles). The productivity of the leading 20 authors, as displayed in Figure 5B, reveals their consistent contributions to the field with a regular publication of articles each year, showcasing their unwavering commitment to this research domain. Notably, WANG J has been publishing articles in this field since 2005, demonstrating a long-term commitment to research. To identify key authors and investigate collaboration patterns, a visualization of the collaboration network was conducted using VOSviewer, as shown in Figure 5C. For enhanced clarity, the collaboration network focused on displaying the collaboration among 116 authors who had contributed to at least 10 papers. The network revealed close collaboration among the authors, with each author’s centrality being less than 0.1. Within the collaboration network, Dong, Zheng also exhibited the closest collaboration, characterized by a total link strength of 128. Subsequently, isaka, yoshitaka (86) and takabatake, yoshitsugu (20) were also notable collaborators in the network. The co-citation network analysis described the collaboration among 116 authors who had at least 10 papers and received citations of more than 100 times (Figure 5D). The co-citation analysis also indicated that the top three authors were Dong, Zheng, isaka, yoshitaka (86), and takabatake, yoshitsugu. Isaka.

### 3.5 Keyword and hotspots analysis

**FIGURE 6:**
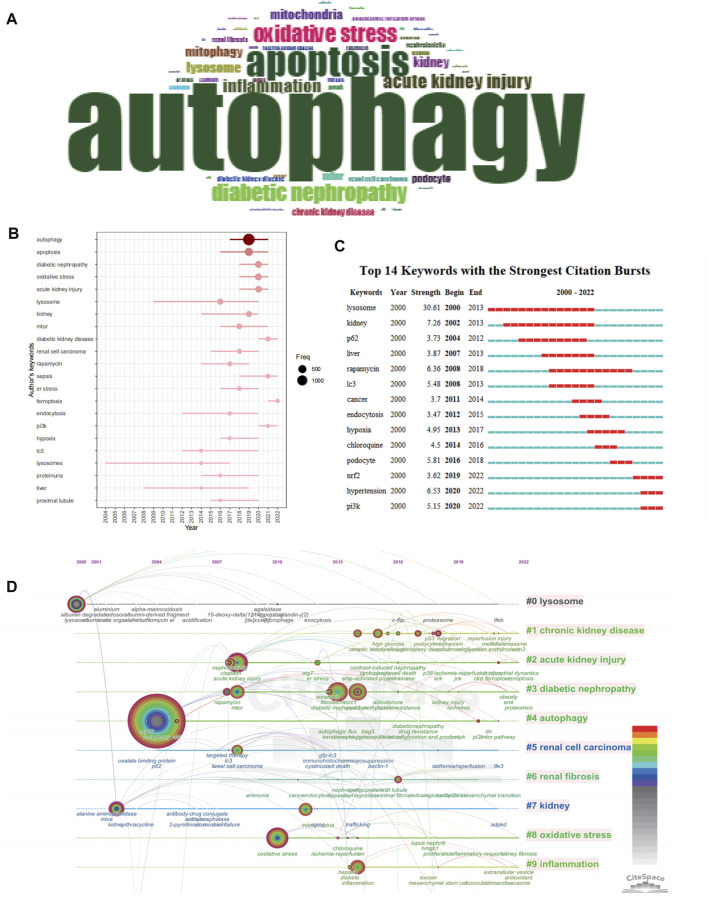
Keyword occurrence and burst analysis. **(A)** Word cloud based on author's keyword by using R-bibliometric. **(B)** Trend topics based on author's keyword over time by using R-bibliometric. **(C)** The top 14 author's keywords with the strongest citation bursts by using citespace. **(D)** The timeline view of author's keywords by using citespace.

In the 4,512 literature articles related to autophagy and kidney diseases, a comprehensive keyword analysis was conducted using the “Author Keywords” in the biblioshiny application and the “Keywords Plus” provided by the Thomson Reuters editorial team. A grand total of 7,348 keywords were identified. However, upon comparing the two sources, it became evident that the “Author Keywords” offered more accurate outcomes, making them the primary data for analysis. The frequency of each keyword was visually represented using a word cloud, as shown in Figure 6A. The term “autophagy” appeared most frequently, being mentioned 1,494 times, followed by “apoptosis” (504 times) and “oxidative stress” (276 times). Notably, recent years have witnessed the emergence of high-frequency keywords such as “diabetic kidney diseases”, “sepsis”, “ferroptosis”, and “pi3k" (Figure 6B). The keyword burst map by CiteSpace also reveals a shifting focus of research towards the following themes: “NRF2″, “PI3K” and “HYPETENSION” (Figure 6C). The timeline visualization of keyword clustering is illustrated in Figure 6D, highlighting the research hotspots of recent years, the hotspots including disease areas such as acute kidney injury, diabetic kidney disease, and chronic kidney disease, as well as mechanism-related hotspots like oxidative stress and inflammation.

**FIGURE 7:**
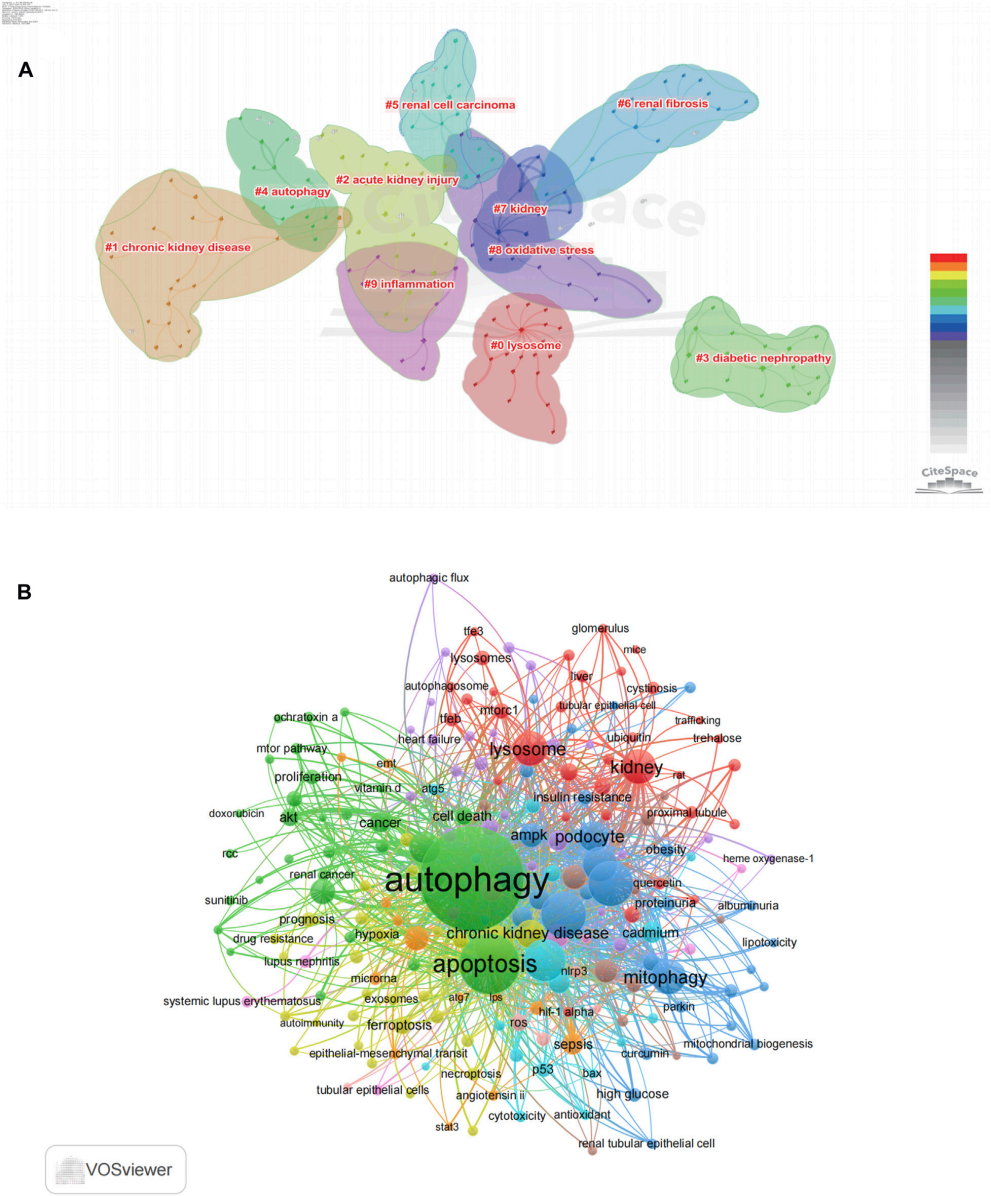
Co-occurrence analysis of author's keywords. **(A)** The co-occurrence clusters of author's keyword by using citespace. **(B)** The co-occurrence visualization network of 205 keywords with a frequency of more than 10 times by using VOSviewer.

We conducted keyword analysis of the 4,579 articles using VOSviewer and CiteSpace software. Co-occurrence analysis was performed to study the connections between keywords and identify popular themes. This method assists researchers in gaining a deeper comprehension of scientific advancements within research hotspots by clustering keywords based on their co-occurrence. In CiteSpace, we found that these keywords could be clustered into 14 categories, including chronic kidney disease, acute kidney injury, diabetic nephropathy, autophagy, renal cell carcinoma, renal fibrosis, kidney, oxidative stress, inflammation, and lysosome (Figure 7A). Similarly, in VOSviewer, we selected the top 205 keywords with at least 10 occurrences to establish a co-occurrence network (Figure 7B) and identified four clusters represented by different colors (red, green, blue, and yellow). Nodes sharing the same color within a cluster indicate closely related co-occurrence, and the size of the nodes and the width of the links vary based on the degree and strength of co-occurrence. The betweenness centrality is applied to gauge the significance of nodes in the network, with larger nodes and higher total link strength indicating a greater amount of information conveyed. The largest node with the highest total link strength in the red cluster is “Autophagy,” which is the main focus of our study. Through in-depth analysis of these four clusters, we observed that each cluster appears to be associated with specific diseases and pathways. The red cluster seems to be more inclined towards the study of autophagy-related mechanisms such as mtor, akt, and apoptosis, while the green cluster is associated with lysosome which mentions lysosome, TFEB, and mTORC1. The yellow cluster focuses on research associated with CKD, with keywords such as chronic kidney disease, fibrosis and epithelial-mesenchymal transit. The dark blue cluster is associated with DKD, represented by keywords such as oxidative stress, mitophagy, and diabetic kidney disease. The light blue metions AKI, including acute kidney injury, reactive oxygen species and cytotoxicity.

## 4 Discussion

Over the past years, the significance of autophagy in the development of kidney diseases has garnered growing interest, positioning autophagy as a prominent research focus in the field of kidney disease. This research marks the initial endeavor to employ bibliometric analysis to examine publications on autophagy and kidney diseases. We performed bibliometric visualization analysis using R software, VOSviewer and CiteSpace on 4,579 literature articles related to autophagy and kidney diseases from the WoSCC database. The outcomes of this study furnish a thorough outline of the research hotspots pertaining to autophagy and kidney diseases during 2000–2022. According to publication output trends, the count of articles associated with autophagy and kidney diseases has gradually increased since 2007. In particular, we have observed explosive growth in the field of autophagy following its recognition with the Nobel Prize in 2016.

Furthermore, our findings emphasize the favorable influence of open access publishing in advancing research within the domain of autophagy and kidney diseases. Open access articles not only demonstrate higher publication growth rates but also attract more citations, indicating their broader influence and potential for collaboration and knowledge dissemination. These results emphasize the importance of data availability and accessibility in promoting scientific progress and advancing research in the field of autophagy and kidney diseases.

Journal publication analysis uncovers a notable enthusiasm for exploring the function of autophagy in kidney diseases from renowned journals like the journal of the american society of nephrology, kidney international, and the specialized journal autophagy. This indicates the robust vitality of this research direction, indirectly driven by renowned journals’ interest. China and the United States are the major research countries in this field, with China being the leading country in terms of research output. This indicates that autophagy has garnered increasing attention in Chinese research institutions in the kidney diseases field. Nevertheless, there is still a certain difference in the impact of publications between China and the United States, implying that China’s overall quality of publications is relatively lower to the United States. Therefore, China needs to produce more high-quality research. Additionally, extensive collaborations have been established among the United States, China, Japan, Germany, South Korea, and other countries. Among the 20 most prominent research institutions, 15 are located in China, including Nanjing Medical University, Shandong University, China Medical University and etc. Other countries such as Osaka University in Japan, the University of Pittsburgh and the University of Pennsylvania in the United States, the University of Texas Southwestern Medical Center Dallas in Germany, and Catholic University of Korea in South Korea are also top 20 prolific institutions. Their significant outputs showcase their active involvement and impact in advancing research. Currently, the global allocation of research in this field is unbalanced. Although Chinese research institutions have rapidly developed and occupied a prominent position, it is imperative to enhance cooperation and scholarly impact among countries and institutions. Based on author analysis, Dong, Zheng emerges as the most productive researcher, having actively collaborated with numerous researchers. Dong, Zheng has dedicated extensive research to investigating the function of autophagy in kidney and convincingly proposes that autophagy plays a pivotal cellular process in maintaining kidney health and function. (Tang et al., 2020). In AKI, Dong, Zheng’s research demonstrated that in ischemia-reperfusion and cisplatin induced mouse models, inhibiting autophagic flux either pharmacologically or genetically led to aggravated renal damage, providing confirmation of autophagy’s protective role in AKI(Periyasamy-Thandavan et al., 2008; Jiang et al., 2012). Furthermore, in diabetic nephropathy (DN), Dong, Zheng also highlights that the downregulation of ULK1 is responsible for autophagy impairment in DN (Ma et al., 2020). Additionally, he also points out the critical involvement of mitophagy in DN and AKI(Xiao et al., 2017; Tang et al., 2018). Another Japanese scholar, Koya, Daisuke, advocates autophagy’s role in regulating metabolic diseases and aging, publishing an article titled “Autophagy in metabolic disease and ageing” in Nature Reviews Endocrinology (Kitada and Koya, 2021). Particularly in DKD, a typical metabolic renal disease. Koya, Daisuke proved that dysfunctional podocyte autophagy worsens proteinuria (Tagawa et al., 2016). In addition, researchers such as Isaka, Yoshitaka, and Takahashi, Atsushi in the purple cluster highlight the close relationship between autophagy and renal inflammation. Excessive inflammation can be cleared through autophagy, demonstrating the tremendous potential of autophagy as a therapeutic approach in regulating renal injury and inflammation (Kimura et al., 2017). The aforementioned authors have made outstanding contributions by offering extensive knowledge about autophagy in kidney diseases and holds promise for future medical treatments.

**FIGURE 8:**
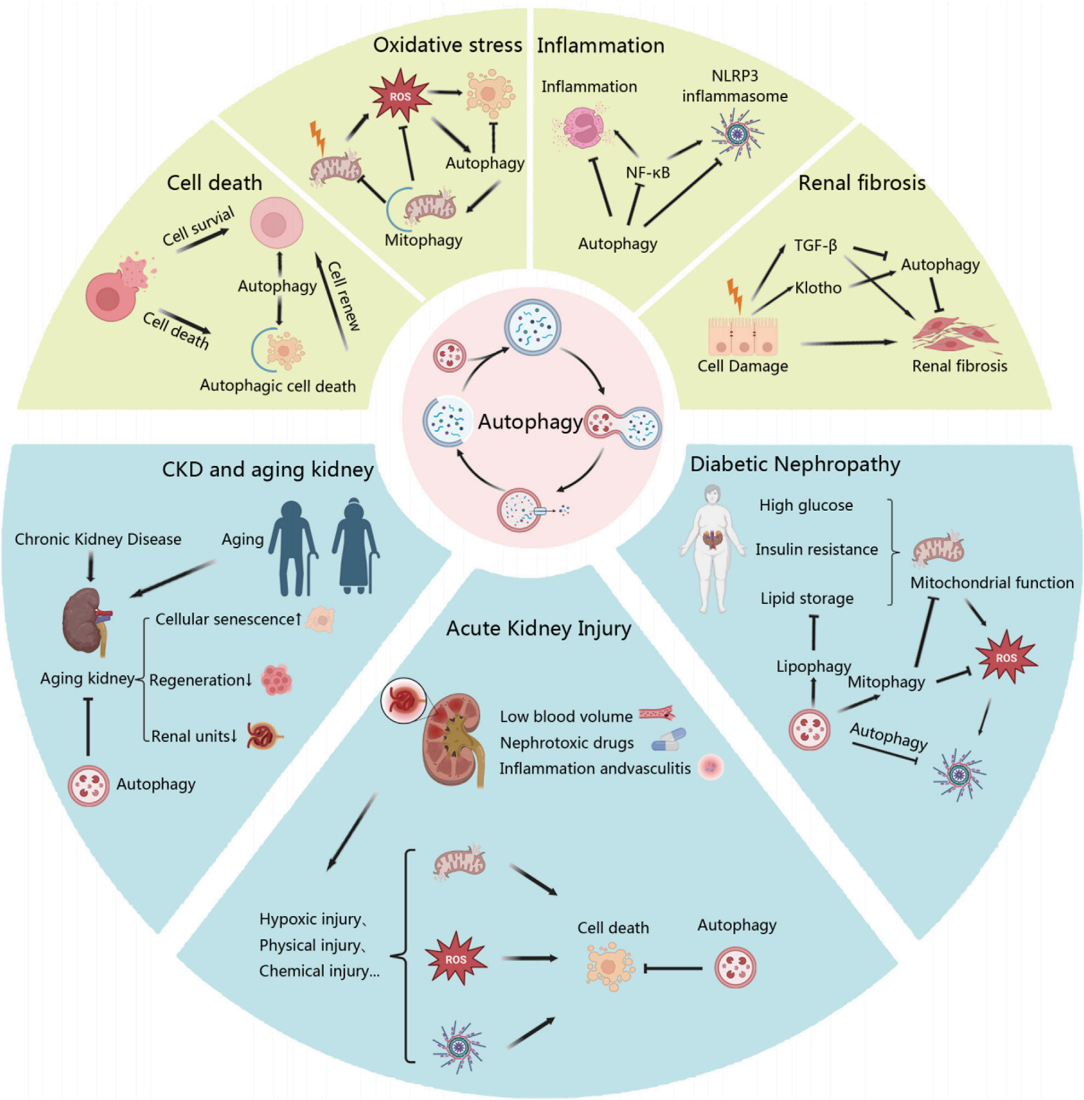
Key mechanisms in autophagy’s regulation in kidney diseases based on keywords.

Based on the current analysis of keywords, the research hotspots in autophagy and kidney diseases mainly revolve around AKI, DKD, and CKD. In different kidney diseases, autophagy may exert various roles, like anti-inflammatory, antioxidant, and anti-aging effects. In this paper, we will delve into the detailed examination of autophagy’s involvement and key mechanisms in these diseases, as shown in Figure 8.

### 4.1 The regulation mechanisms of autophagy

Autophagy plays a vital role in kidney diseases, manifesting in diverse renal cell types, including podocytes, tubular cells, mesangial cells, and endothelial cells (Liu et al., 2014; Xu et al., 2016; Bork et al., 2020; Liang et al., 2020). Depending on the mechanism of autophagic degradation, autophagy can be categorized as macroautophagy, microautophagy, or chaperone-mediated autophagy (Tang et al., 2020; Wang et al., 2023a). Additionally, autophagy can also involve the selective degradation of specific substrates, such as mitophagy and lipophagy (Li W. et al., 2021). Currently, macroautophagy, commonly mentioned as autophagy, is the typical studied form. The process of autophagy begins with the regulation of autophagy-related genes (ATGs) and Beclin-1. The generation of autophagosomes, double-membraned vesicles, is initiated under the regulation of these factors. Autophagy cargo is sequestered within these autophagosomes through the involvement of proteins like P62. Subsequently, the autophagosomes fuse with lysosomes, forming autolysosomes. Lysosomes contain hydrolytic enzymes and an acidic environment, facilitating the degradation of autophagic cargo (Dikic and Elazar, 2018).

Autophagy is regulated by various factors. Firstly, the mTOR functions as a suppressor of autophagy (Shimobayashi and Hall, 2014; Saxton and Sabatini, 2017). MTOR, along with regulatory-associated protein, forms complexes mTORC1, which phosphorylates and inhibits Unc-51 Like autophagy activating kinase 1(ULK1) and ATG13, thus preventing autophagy initiation (Dossou and Basu, 2019). ULK1 is one of the crucial factors in regulating the autophagy pathway, and activated ULK1 phosphorylates multiple substrates, including Beclin 1, to facilitate autophagosome formation (Russell et al., 2013). And ULK1 is also inhibited by mTOR, preventing autophagy initiation. MTORC1 also regulates lysosome biogenesis and function through the phosphorylation and inhibition of transcription factor EB (TFEB), ensuring the smooth flow of autophagy (Cui et al., 2023). MTORC1 is subject to regulation by numerous signaling pathways within the cellular environment. Another common regulator is AMPK, which phosphorylates and activates ULK1, thereby directly inducing autophagy (Kim et al., 2011). AMPK also indirectly regulates autophagy activity by activating the tuberous sclerosis complex 1 (TSC1)-TSC2 complex and inhibiting Rheb, thereby suppressing mTORC1 activity (Gwinn et al., 2008). What’s more, the phosphatidylinositol 3 kinase/protein kinase B(PI3K/AKT) pathway also participates in the regulation of autophagy. PI3K catalyzes the phosphorylation of phosphatidylinositol (PI) on the cell membrane to form phosphatidylinositol-3,4,5-trisphosphate (PIP3). As a second messenger, PIP3 activates AKT. Activated AKT phosphorylates TSC2, directly inhibiting the inhibitory effect of the TSC1-TSC2 complex on Rheb and thus enhancing mTORC1 activity (Cantley, 2002; Inoki et al., 2002; Inoki et al., 2003; Fruman et al., 2017). Additionally, based on keyword analysis, SIRTs are identified as key regulatory molecules in autophagy. SIRTs, as a class of nicotinamide adenine dinucleotide (NAD^+^)-dependent deacetylases, exert their function on autophagy by modulating the deacetylation of multiple proteins (Lee et al., 2008; Carafa et al., 2016). SIRTs, including SIRT1, SIRT3, SIRT4, and SIRT6, can directly or indirectly control the function of several autophagy-related transcription factors (Zhang et al., 2019; Zhang et al., 2020; Wang L. et al., 2021; Xu et al., 2021; Kim et al., 2022; Li et al., 2023). Among them, SIRT1 has been the subject of extensive study. SIRT1 enhances the transcriptional activity of factor forkhead box O (FOXO) through deacetylation, thereby stimulating the transcription of autophagy-related genes (Hariharan et al., 2010; Kume et al., 2010; Nath, 2010).

The analysis reveals that lysosomes are a major keyword associated with autophagy and kidney diseases. Lysosomes are a crucial component of the autophagy process, and their proper formation and function ensure the smooth flow of autophagy (Festa et al., 2018). Lysosomal dysfunction can lead to cell death. Lysosomal membrane permeabilization (LMP) results in the release of tissue proteases and other hydrolytic enzymes from the lysosomal lumen into the cytosol, which has been shown to initiate cell death pathways in specific circumstances (Serrano-Puebla and Boya, 2016). The modulation of lysosomes primarily involves various signaling pathways and protein factors. Among them, TFEB is an established central regulator of the lysosomes (Tan et al., 2022). Enhancing the activity of TFEB is crucial for promoting lysosomal biogenesis and maintaining lysosomal function. The activity of TFEB is regulated by various mechanisms, including mTORC1, AMPK, and Ca^2+ signaling pathways (Cao et al., 2020; Park et al., 2022; Cui et al., 2023). Additionally, mucolipin-1 plays a crucial role in inducing TFEB activation. Activating mucolipin-1 promotes lysosomal exocytosis and significantly reduces uranium-induced damage and cell death in renal proximal tubule epithelial cells (Zhong et al., 2023). Shuhei Nakamura demonstrated that lysosomal damage triggers the recruitment of LC3 to lysosomes. Subsequently, lipidated LC3 interacts with the lysosomal calcium channel TRPML1 and promotes the calcium efflux necessary for TFEB activation (Nakamura et al., 2020). Therefore, therapeutic approaches targeting TFEB may help promote renal protection by enhancing the autophagic flux.

Mitophagy is a selective form of autophagy that targets damaged or aged mitochondria. When mitochondria are damaged, phosphatase and tensin homolog (PTEN)-induced putative kinase 1(PINK1) accumulates at the surface of the outer mitochondrial membrane, resulting in the activation of parkin. Activated Parkin directs the binding of proteins associated with autophagosomes to mitochondria, triggering the selective degradation of mitochondria (Koyano et al., 2014; Lazarou et al., 2015; Tang et al., 2018; Onishi et al., 2021). A recent study reported that PINK1-PARK2- optineurin-mediated mitochondrial autophagy is activated and plays a protective role in septic acute kidney injury (Wang Y. et al., 2021). In addition to PINK1-parkin-mediated mitophagy, BCL2/adenovirus E1B 19 kDa protein-interacting protein 3(BNIP3) can also mediate mitochondrial autophagy, as one study demonstrated Hypoxia-inducible factor 1α(HIF1α)/BNIP3-mediated mitophagy in tubular cells protects against renal ischemia/reperfusion injury (Fu et al., 2020). Furthermore, a study found that tumor necrosis factor alpha-induced protein 8-like 1 (TIPE1) regulates the ubiquitination and degradation of the mitochondrial autophagy receptor prohibitin 2, disrupting mitochondrial homeostasis, promoting the pathogenesis of diabetic nephropathy (DN), and demonstrating that inhibiting TIPE1 can serve as a novel therapeutic target for DN (Liu L. et al., 2022). Pannexin 1 gene deficiency enhances mitochondrial autophagy and mitigates tubular cell death, oxidative stress, and mitochondrial damage following I/R injury (Su et al., 2023a). This demonstrates that mitochondrial autophagy in the kidney is regulated by multiple mechanisms.

In cases of kidney injury or metabolic disorders, Stagnation of autophagy leads to the accumulation of lipid droplets (Takabatake et al., 2017; Yamamoto et al., 2017). Further LD expansion may occur through fusion or localized lipid synthesis, which can disrupt the normal cellular structure and function (Mitrofanova et al., 2023). Lipophagy is one of the primary pathways for the breakdown of LDs into free fatty acids. A study confirmed a significant lipophagy reduction in autophagic levels in HK-2 cells treated with high glucose, which resulted in lipid droplet accumulation, as well as apoptosis and renal fibrosis in renal cells. (Han Y. et al., 2021). Another study indicates that UCP1 can significantly alleviate lipid accumulation in AKI by promoting the AMPK/ULK1/autophagy pathway, thus inhibiting the progression of AKI(Xiong et al., 2021). These studies demonstrate the existence of lipophagy in cells, and it is considered an emerging field in kidney research.

### 4.2 Autophagy crosstalk with other mechanisms

Based on keyword analysis, it is evident that autophagy exhibits extensive crosstalk and interactions with other cellular mechanisms in kidney diseases. These include cell death, oxidative stress, inflammation, and fibrosis.

One of the closest relationships is observed with cell death, as autophagy exerts a dual regulatory role in this process (Codogno and Meijer, 2005). Autophagy can be activated to clear damaged proteins and organelles while providing vital nutrients for cell viability, thereby protecting proximal tubular cells from injury and apoptosis (Havasi and Dong, 2016). In shock wave-induced renal cell injury, suppressing autophagy exacerbated cell damage or apoptosis, while the activation of the Akt/GSK-3β induced autophagy, serving as a protective mechanism (Long et al., 2016). And autophagy was triggered to repair the reactive oxygen species (ROS)-induced damage and and thereby promote cell survival in AGE-induced mesangial cell damage (Xu et al., 2016). These findings indicate that autophagy exerts protective effects on the kidney by suppressing apoptosis-related pathways. Autophagy can regulate various types of cell death such as ferroptosis and pyroptosis. By enhancing hypoxia-inducible factor 1α (HIF1A) and BNIP3-mediated mitophagy, suppressing the nucleotide-binding oligomerization domain-like receptor family pyrin domain-containing 3(NLRP3) inflammasome alleviates pyroptosis in contrast-induced AKI(Lin et al., 2021). Furthermore, our research has identified that ferroptosis has emerged as a forefront area in the study of autophagy and kidney diseases. A study demonstrated that mitophagy can helps counteract cisplatin-induced renal tubular epithelial cell damage through the ROS/Heme oxygenase 1(HO-1)/Glutathione peroxidase 4 (GPX4) signaling pathway, thereby alleviating the occurrence of ferroptosis (Lin et al., 2023). However, another study shows SIRT3 enhances autophagic activation by promoting the AMPK-mTOR pathway and reducing GPX4 levels, thereby inducing ferroptosis (Han et al., 2020). Moreover, Autophagy can also trigger a type of cell death referred to as “autophagic cell death”, characterized by the formation of autophagosomes and the degradation of intracellular components by lysosomes (Kroemer and Levine, 2008; Lieberthal, 2008). A study also suggest that there is a form of autophagy-dependent ferroptosis, which has been validated in human renal tubular epithelial cells and mouse tissues (Hou et al., 2022). In cadmium (Cd)-induced renal injury, the activation of ferroptosis by ROS requires autophagy, as inhibition of autophagy by chloroquine can alleviate Cd-induced ferroptosis (Zhao et al., 2021). These new findings indicate that there is a complex interaction between autophagy and ferroptosis.

The molecular crosstalk between autophagy and oxidative stress primarily involves ROS-triggered induction of autophagy and autophagy-mediated alleviation of oxidative stress (Jha et al., 2016; Kaushal et al., 2019). Autophagy plays a crucial role in preserving intracellular redox balance by eliminating oxidatively damaged mitochondria while restraining the excessive production of ROS. The relationship between mitochondrial damage, ROS production, and mitophagy defection is intricately intertwined, with all of these processes contributing to kidney injury (Aranda-Rivera et al., 2021). In AKI, damaged mitochondria can produce a significant amount of ROS, which signal for their own clearance through mitophagy, reducing ROS production and forming a redox signaling negative feedback loop (Su et al., 2023b). A study indicates that PINK1-Parkin-mediated mitophagy controls mitochondrial quality and mitochondrial ROS by degrading damaged mitochondria, thereby exerting a protective role in contrast-induced AKI(Lin et al., 2019). Similarly, another study suggests that mild oxidative stress triggered mitophagy thus governing the selective removal of dysfunctional mitochondria (Scherz-Shouval and Elazar, 2007; Frank et al., 2012). These research findings collectively indicate that under specific conditions, autophagy and oxidative stress synergistically regulate the redox signaling balance. Our research results suggest that the Nuclear factor erythroid 2-related factor 2(NRF2)-related signaling pathways may be a current and future research focus. It is widely acknowledged that NRF2 is closely associated with the regulation of redox signaling. Research indicates that autophagy’s role in maintaining redox homeostasis depends on the p62/Kelch-like ECH-associated protein 1(KEAP1)/NRF2 signaling pathway. Typical autophagic cargo proteins and the autophagic substrate p62 can directly interact with KEAP1, competing with the transcription factor NRF2, which is associated with antioxidant responses (Komatsu et al., 2010). Another study further confirms that Keap1 can reversibly translocate to p62-gels in a p62-binding-dependent manner to activate the transcription factor Nrf2. Mice lacking Atg8-dependent selective autophagy show impaired turnover of p62-gels, leading to excessive Nrf2 activation in the body, causing oxidative stress responses (Kageyama et al., 2021).

Regulating inflammation through autophagy holds significant potential for treating damaged kidneys. Increasing evidence suggests that autophagy protects the kidneys from various types of renal inflammatory injuries, including acute, chronic, and metabolic damage (Kimura et al., 2017). In the unilateral ureteric obstruction model, the deletion of tubular epithelial ATG5 genes significantly inhibited autophagy, which led to heightened nuclear factor κB (NF-κB) activation, increased proinflammatory cytokines in the obstructed kidneys (Peng et al., 2019). Additionally, autophagy interacts with NLR family pyrin domain containing 3(NLRP3) inflammasomes, regulating the body’s inflammatory responses. One study indicates that palmitoylation restricts NLRP3 inflammasome activation through autophagy mediated by molecular chaperones, thus preventing sustained inflammation (Wang et al., 2023b). Conversely, inflammatory response can inhibit autophagy, thereby affecting autophagy function. In HFD/STZ-induced DN mice, activating the NLRP3 inflammasome worsens podocyte autophagy and decreases podocyte nephrin expression, while silencing NLRP3 effectively reverses podocyte autophagy and improves podocyte injury caused by high glucose levels (Hou et al., 2020). Another study also suggests that in podocyte-specific NLRP3 inflammasome activation in DKD mice, there is a decrease in autophagy marker LC3II/LC3I ratio and a significant increase in p62 levels, indicating impaired autophagy (Shahzad et al., 2022).

Autophagy regulation may present a promising new approach to delay the progression of kidney fibrosis (Shi et al., 2022). The deficiency of the ATG5 gene also led to increased collagen type 1(COL1) production, further promoting renal fibrosis in AGT II-stimulated primary proximal tubular cells. (Li et al., 2016). Furthermore, in TGF-β1 intervention or the establishment of a UUO model, the elevated expression of RAGE activates STAT3, which directly binds to the promoter region of Atg7, leading to the upregulation of Atg7 and increased autophagy. This promotes the progression of renal fibrosis (Liu B. et al., 2022). In the same way, autophagic degradation also regulate mature TGF-β1 protein levels in renal tubular epithelial cells, effectively suppressing kidney fibrosis caused by unilateral ureteral obstruction (Ding et al., 2014). All of this evidence collectively indicates that the downregulation of autophagy contributes to the progression of renal fibrosis. Moreover, in the DOCA-salt-sensitive hypertensive mouse model, adiporon promotes autophagy by activating the AMPK signaling pathway, thereby inhibiting the epithelial-mesenchymal transition (EMT) of renal tubular epithelium (Li Y. et al., 2021). Additionally, research has confirmed that the expression of endogenous Beclin 1 protein in renal tubules and the expression of exogenous Beclin 1 peptide can promote the recovery of the kidneys after AKI by inhibiting renal fibrosis (Shi et al., 2022). These studies demonstrate that autophagy serves as an anti-fibrosis mechanism by promoting cell survival and facilitating repair.

### 4.3 Autophagy and kidney disease

Through keyword analysis, we have identified that research on autophagy and kidney disease mainly focuses on diabetic nephropathy, acute kidney injury, and chronic kidney disease.

There is a close relationship between autophagy and DKD (Yamahara et al., 2013; Kume and Koya, 2015). Keyword analysis reveals that the association between autophagy and DKD primarily centers around mitochondrial autophagy, oxidative stress, and lipid metabolism. This suggests that autophagy in DKD is strongly associated with these themes. DKD is closely associated with nutrient sensing, and nutrients act as a switch for autophagy (Kume et al., 2012). Research has indicated that autophagy in renal podocytes and endothelial cells can alleviate insulin resistance, protect against high glucose damage, and reduce glomerulosclerosis in diabetic nephropathy (DN) (Lenoir et al., 2015; Xin et al., 2016). In DKD patients, high blood glucose and metabolic abnormalities impair mitochondrial function (Khoshjou and Dadras, 2014). Mitochondrial damage causes increased reactive oxygen species (ROS), and mitophagy is a process that promotes the self-renewal of damaged mitochondria and reduces the levels of ROS in diabetes (Khoshjou and Dadras, 2014). Excessive ROS can damage cell membranes, DNA, and proteins, resulting in oxidative damage in renal tissues, triggering cell apoptosis and leading to renal cell death (Bandyopadhyay et al., 1999; Sifuentes-Franco et al., 2018). Oxidative stress activates also mediated NF-κB and NLRP3 inflammsome activation, futher promoting inflammatory responses in DN (Zhou et al., 2018; Hu et al., 2021). Enhancing mitochondrial autophagy help reduce ROS production, and protect the kidney from further damage caused by ROS(Yun et al., 2020). Study have shown that antioxidant can exerts positive impacts beneficial effects on tubular injury in DKD by promoting Nrf2/PINK-mediated mitochondrial autophagy (Xiao et al., 2017). Various drugs have been observed to be associated with the modulation of autophagy in the treatment of DKD. Metformin is a well-established AMPK agonist that can induce mitophagy, thereby alleviating renal tubulointerstitial fibrosis in a high-fat diet and streptozotocin-induced mice (Han Y. C. et al., 2021). And the effects of sodium-glucose cotransporter-2 (SGLT2) inhibitors may be related to enhanced SIRT1 and HIF-2α signaling, which exert renoprotective effects by promoting autophagic flux (Packer, 2020). Due to energy excess and mitochondrial damage, lipid metabolism disorders occur, which seems to be a research focus in recent years regarding DKD (Opazo-Ríos et al., 2020; Thongnak et al., 2020; Mitrofanova et al., 2021). In high-fat diet mice, disrupted lysosomal function and blocked autophagic flux is observed, aggravating lipotoxicity in the kidney (Yamamoto et al., 2017). Additionally, lipophagy deficiency is proved in in DKD and exacerbates the accumulation of lipids and injury to tubular cells (Han Y. et al., 2021)

Through keyword analysis, we have found that in the cluster of AKI, key terms include cellular necrosis, endoplasmic reticulum stress, Bcl-2, Beclin 1 and etc. These results show a significant interactions autophagy and cell death in AKI. Cell death is a crucial pathological characteristic in AKI, which can be triggered by multiple factors, such as ischemia-reperfusion injury, drug or toxin exposure, and infection. These stimuli can lead to cellular stress responses in renal cells, including oxidative stress, intracellular calcium imbalance, and mitochondrial dysfunction, thus trigger cell necrosis. Interestingly, specific apoptosis-regulating proteins Bcl-2 can bind to Beclin 1, acting as an autophagy regulatory factor (Fernández Á et al., 2018; Prerna and Dubey, 2022). Studys have demonstrated that autophagy serves a protective function in AKI, as evidenced in experimental models of ischemia-reperfusion injury and cisplatin-induced AKI(Jiang et al., 2010; Jiang et al., 2012). Autophagy is capable of eliminating oxidative stress and inflammatory factors, thereby inhibiting cell apoptosis protects the kidney from further injury. Moreover, autophagy provides energy and metabolic substances, maintaining normal cell function and promoting kidney repair and recovery (Kaushal and Shah, 2016).

CKD is a progressive condition characterized by a gradual decline in kidney function and reduced glomerular filtration rate along with renal cell dysfunction, structural changes, and tissue fibrosis. Renal fibrosis represents a crucial characteristic of disease progression in CKD. It involves the buildup of extracellular matrix (ECM) and stimulation of fibroblasts (Humphreys, 2018). Autophagy plays a role in preventing renal fibrosis. In the unilateral ureteral obstruction experimental model, autophagy controlled the progression of fibrosis by regulating key fibrosis-related molecule TGF-β(Ding et al., 2014). Furthermore, in kidney disease, there is a significant association between autophagy and ageing (Hartleben et al., 2010; Huber et al., 2012; Fougeray and Pallet, 2015; O'Sullivan et al., 2017; Schmitt and Melk, 2017; Minami et al., 2023a). In aging mice, Atg5 gene deletion in podocyte resulted in podocyte loss and the development of glomerulosclerosis (Hartleben et al., 2010). The main features of renal aging are the decline in regenerative potential, cellular senescence, and loss of nephron. Autophagy, by eliminating senescent cells, can weaken cellular aging and enhance kidney vitality (Schmitt and Melk, 2017)。Additionally, α-klotho is a membrane-bound protein highly expressed in the kidneys and closely linked to the aging process. Mouse klotho gene mutations lead to a syndrome that resembles aging (Kuro-o et al., 1997). Research has shown that klotho promotes autophagy to alleviate kidney inflammation and impact the progression of kidney diseases (Zhu et al., 2021). These findings show that the crucial regulatory function of autophagy in the kidney aging process, promoting cell regeneration and repair. In a 24-month aging model, it has been demonstrated that a strong induction of FGF 21 is provoked by autophagy deficiency to prevent the progression of CKD under aging conditions. The lack of FGF 21 exacerbates lysosome overload and worsens autophagic stagnation (Minami et al., 2023b). What’s more, a article published in Nature has discovered that through Beclin1 mutations, releasing Beclin1 from the negative regulator BCL2 plays a regulatory role in autophagy. This mechanism could be an effective way for mammals to enhance cellular autophagy, prevent premature aging, and promote longevity (Fernández Á et al., 2018). These evidences presented above collectively demonstrate a close relationship between autophagy and kidney aging.

## 5 Limitation

In contrast to conventional review articles, we using to offer a comprehensive and quantified analysis of research emphases, patterns, and collaborative efforts in the field of autophagy in kidney diseases, allowing for better understanding of the field’s progression. Nevertheless, it is important to acknowledge certain limitations in our study. Firstly, due to the limitations of the software, we focused solely on publications from the WOSCC database, excluding non-English publications, which may introduce publication bias. Secondly, it's important to consider that citation data is influenced by time, with older papers often receiving more citations than newer ones. As a result, some prominent papers might not gain the recognition they deserve due to their relatively shorter period of availability. Additionally, the prevalence of citing review articles over original research may also introduce bias. Furthermore, using different software combinations for literature analysis may lead to the omission of information during the analysis process, resulting in minor differences in the results.

In addition to the limitations in our analytical methods, our discussions on key topics have revealed certain constraints in the current state of autophagy research. Firstly, our analysis indicates that the focus of autophagy research has largely remained on conventional areas such as inflammation, oxidative stress, and kidney fibrosis. However, our analysis has also unveiled some newer research areas like aging, NRF2, ferroptosis, and lipid autophagy, which may become future research hotspots. Furthermore, when it comes to kidney disease research, the majority of studies have been concentrated on AKI, CKD and DKD. Other kidney diseases such as lupus nephritis, membranous nephropathy, and IgA nephropathy have received relatively less attention. Future research should aim to explore the specific roles of autophagy in these different types of kidney diseases. Additionally, we have noticed a significant scarcity of research on autophagy in the context of kidney disease treatment. Most studies have been centered on elucidating mechanisms, with relatively few focusing on therapeutic interventions related to autophagy. This suggests that, while autophagy has become a consensus in research, there is a lack of targeted drugs that have reached the stage of clinical application. Therefore, it is imperative for autophagy research to accelerate its translation towards clinical applications. Although our study has certain limitations, we remain confident that it offers a thorough investigation of autophagy in kidney diseases, delivering valuable perspectives for both present and future research endeavors in this area.

## 6 Conclusion

This pioneering study offers a thorough analysis of the research direction and upcoming prospects of autophagy in kidney diseases. Overall, there has been a rapid growth in the volume of publications within this research domain during 2000–2022. China has emerged with a significant level of productivity in this specific research field, while the United States stands as the most influential country. Strengthened global collaboration between China and the United States is warranted to conduct more extensive research in this field. Journal analysis reveals that autophagy research in kidney diseases is a focal point in kidney disease research, with influential and highly productive journals in the field, such as journal of the american society of nephrology kidney international, driving its development. Keyword clustering analysis indicates that research in this field primarily focuses on diseases like CKD, AKI, and DKD, particularly the interaction between autophagy and regulatory cell death, oxidative stress, inflammation, and mitochondria being key areas of investigation. Current frontiers in disease research primarily revolve around diabetic kidney disease and hypertension. Cutting-edge research in understanding the mechanisms involves areas such as PI3K, NRF2, and ferroptosis. These timely analytical results offer new perspectives for therapeutic approaches in managing kidney diseases, and assist researchers in selecting appropriate journals for publication, identify potential collaborators, and stay informed about hot topics and frontiers, thereby advancing the field.
