# Supplementary figures and images for: Global research trends and hot spots on autophagy and kidney diseases: a bibliometric analysis from 2000 to 2022
Source: Front Pharmacol. 2023 Nov 30;14:1275792. doi: 10.3389/fphar.2023.1275792 (PMC10719858; doi:10.3389/fphar.2023.1275792)

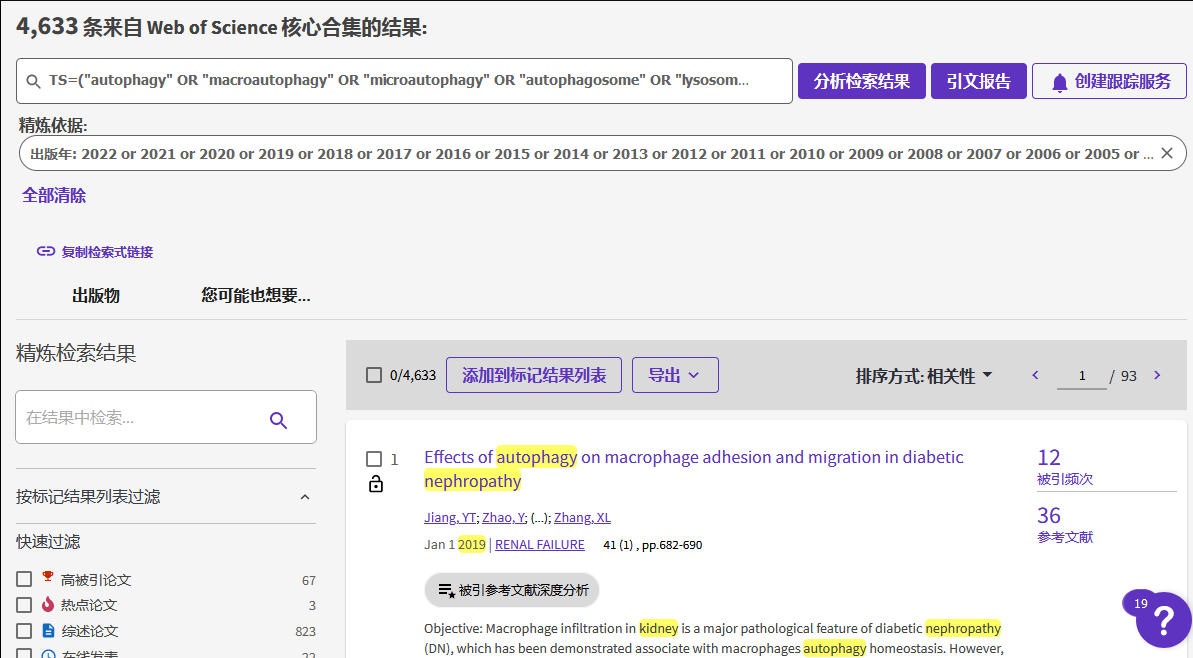

Supplement: Supplementary file 1 [file DataSheet1.ZIP › Raw data/Search result.png]
