# Supplementary material for: Global research trends and hot spots on autophagy and kidney diseases: a bibliometric analysis from 2000 to 2022
Source: Front Pharmacol. 2023 Nov 30;14:1275792. doi: 10.3389/fphar.2023.1275792 (PMC10719858; doi:10.3389/fphar.2023.1275792)
Supplement: Supplementary file 3 [file Image1.pdf]

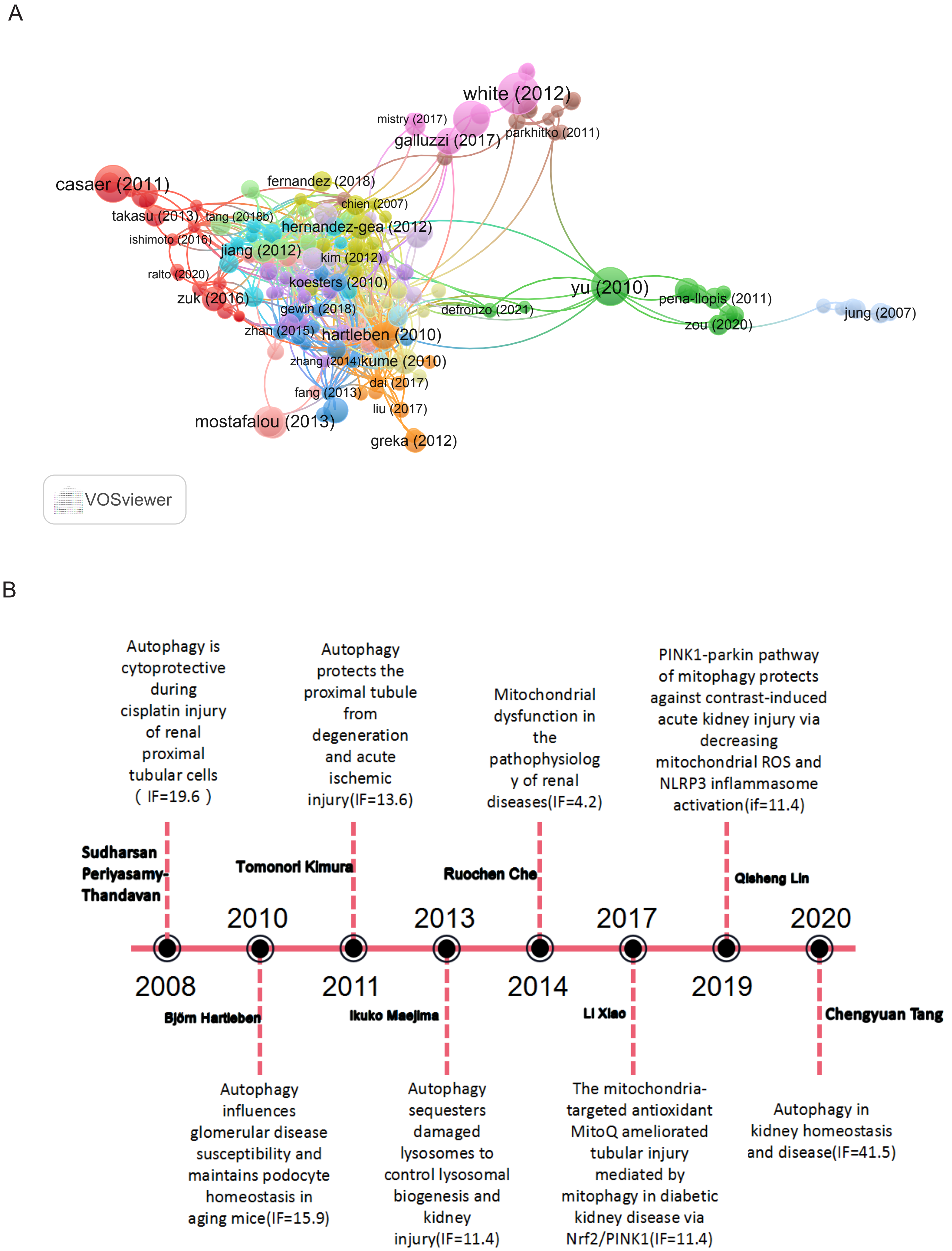

FIGURE S1. Analysis of key articles. (A)Co-citation network analysis of articles by using VOSviewer. (B)Timeline of key articles in autophagy and kidney disease research.
